# Absence of the RET+3:T allele in the MTC patients

**DOI:** 10.1186/1897-4287-10-14

**Published:** 2012-10-22

**Authors:** Pawel Borun, Sowinski Jerzy, Katarzyna Ziemnicka, Lukasz Kubaszewski, Daniel Lipinski, Andrzej Plawski

**Affiliations:** 1 Institute of Human Genetics Polish Academy Sciences, ul. Strzeszyńska 32, 60-479, Poznan, Poland; 2 University of Medical Sciences, Poznan, Poland

## Abstract

The mutations of the RET proto-oncogene contributes to the development of MTC by increasing the activity of the receptor encoded by this gene. Variant T of polymorphism rs2435357 located in the enhancer of the RET gene reduces the enhancer’s activity. The opposite effects of rs2435357 and the mutations causing medullary thyroid carcinoma resulted in the investigation of the status of this polymorphism in patients with MTC. In our study, we compared the frequency of polymorphism rs2435357 in the group of 48 MTC patients with its frequency in Polish population. The frequency of heterozygotes C/T at rs2435357 reached almost 12% (18/152) for the Polish population, in contrast to the group of MTC patients where not even a single T allele was found. The frequency difference is statistically significant. This observation might indicate that the presence of the heterozygous T allele at rs2435357 may be associated with the inhibition of medullary thyroid carcinoma development.

## Background

The RET proto-oncogene (MIM 164 761), located on 10q11.21, encodes a receptor tyrosine kinase. At the end of the 90s, the association of RET gene mutations with the occurrence of medullary thyroid carcinoma (MTC) and Multiple endocrine neoplasia type 2A (MEN 2A) was described [1,2]. Mutations in the RET proto-oncogene, leading to the development of MTC, increase the activity of the receptor encoded by this gene. These mutations occur in the part of gene encompassing exons from 11 to 16, and 75–80% of all cases of hereditary MTC carry a mutation in codon 634 located in exon 11 [3].

The common polymorphism rs2435357 (previously termed RET+3:C/T, with an ancestral allele C and a derived mutation allele T) in the noncoding enhancer element MCS+9.7 (Multispecies Conserved Sequence located 9.7 kb from the ATG codon) in intron 1 of the RET gene is a HSCR (Hirschsprung Disease) susceptibility allele [4,5]. A single mutation allele T is associated with 6-fold decrease in activity of the RET gene enhancer. The homozygous variant T/T increases the risk of Hirschprung disease 20-fold [5]. Variation in the frequency of allele T is observed in the world. Scientific data shows that it is virtually absent within Africa, and has an intermediate frequency in Europe (0.25), however it reaches high frequency (0.45) in Asia [4,5].

The RET gene mutations, leading to the occurrence of MTC, increasing the protein activity and variant T at rs2435357 acts, in a sense, antagonistically to the RET gene mutations, therefore it was interesting to investigate the prevalence of this allele in our 48-person group of patients with medullary thyroid carcinoma.

## Methods

**Figure 1:**
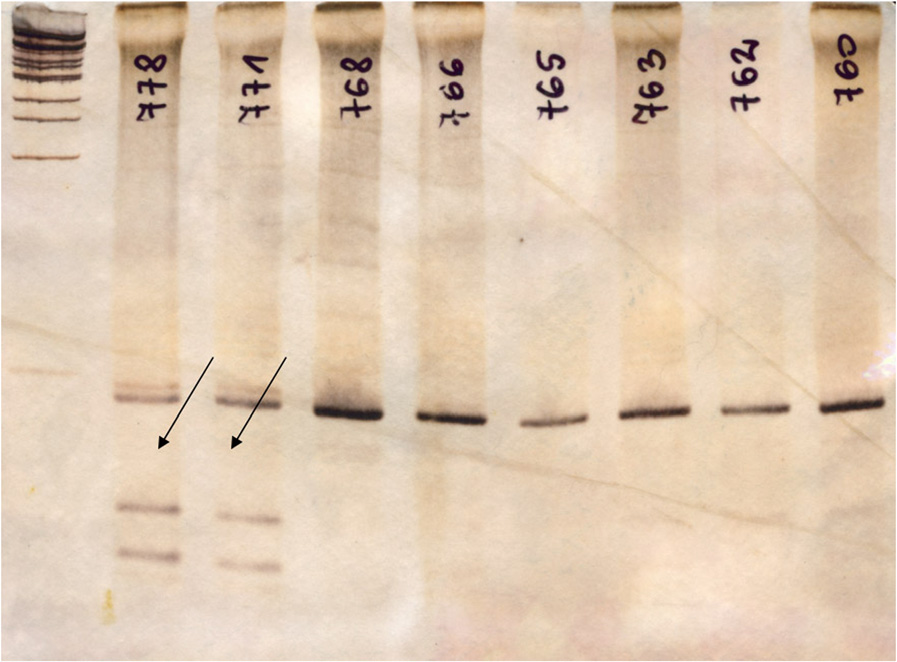
**Restriction analysis of the fragment of *RET *gene for the control group.** Arrows indicates the digested RET+3:T allele. The separation was performed on 6% polyacrylamide gel

**Figure 2:**
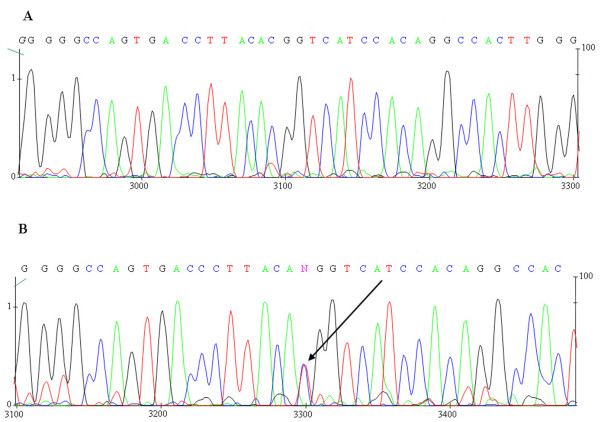
**Sequence analysis of the fragment of the RET gene encompassing C/T polymorphism.** Arrow indicates the polymorphic site. Direct sequencing of PCR product performed using DYEnamic ET dye terminator (GE Healthcare). The sequencing reaction products were analyzed on MEGABACE 500 DNA sequencer (GE Healthcare)

The group of 48 patients (37 men and 11 women) with medullary thyroid carcinoma participating in the study was not screened for mutations in the RET gene and was selected on the basis of clinical symptoms. In 4 cases, a family history of medullary thyroid carcinoma (FMTC) was reported. The control group consisted of 152 anonymous individuals (76 men and 76 women) randomly selected from the Polish population. The analysis of alleles frequency at rs2435357 was performed by using the PCR-RFLP method. In the first stage, the RET gene fragment containing rs2435357 was amplified using forward primer 5^′^gagtgcatggggacagtt3^′^ and reverse primer 5^′^ggaaactgccaattaggttat3^′^. Subsequently, the PCR product was subjected to restriction digestion with endonuclease Hin1II. The T allele creates a restriction site for the enzyme. The digestion products were separated on 6% polyacrylamide gel and afterwards DNA products were visualized with silver staining (Figure 1). The occurrence of the T alleles detected in the analysis was confirmed by sequencing (Figure 2). The studies was approved the local Ethics Committee at the Poznań University of Medical Science, resolution No. 629/07. Written informed consent was obtained from the patient for publication of this report and any accompanying images.

## Results and discussion

For the Polish population, the incidence of heterozygous variant rs2435357 reached almost 12% (18/152), while in the study group of patients with MTC not even a single T allele was found (Table 1). Frequency difference is statistically significant and according to the Fisher’s Exact Test the two-sided P value is 0.0080.

**Table 1:** Frequency of the rs2435357 polymorphism in the studied group

	**Woman**		**Man**	
	**rs2435357 C/C**	**rs2435357 C/T**	**rs2435357 C/C**	**rs2435357 C/T**
MTC patients	37	0	11	0
Control	68	8	66	10

Mutation allele T is associated with reducing the activity of the RET gene enhancer. The consequences of this reduction have a negative impact on homozygotes by increasing the risk of Hirschprung disease several dozen times. The obtained results led to the conclusion that possibly the presence of heterozygous mutant allele T at rs2435357 can inhibit the development of medullary thyroid carcinoma. This may be related to the antagonistic effects of this polymorphism in relation to the mutations that increase the activity of the protein causing MTC. This conclusion was drawn based only on a study of a small group of patients with MTC and should be confirmed in a larger cohort.

Taking our observation into consideration, it would be interesting to investigate the possible role of the rs2435357 in modifying the course of MEN2A.
